# Identification of circulating tumor cells captured by the FDA-cleared Parsortix^®^ PC1 system from the peripheral blood of metastatic breast cancer patients using immunofluorescence and cytopathological evaluations

**DOI:** 10.1186/s13046-024-03149-x

**Published:** 2024-08-21

**Authors:** Mariacristina Ciccioli, Kyukwang Kim, Negar Khazan, Joseph D Khoury, Martin J Cooke, M Craig Miller, Daniel J O’Shannessy, Anne-Sophie Pailhes-Jimenez, Richard G Moore

**Affiliations:** 1 ANGLE Europe Limited, Guildford, UK; 2 Division of Gynecologic Oncology, Department of Obstetrics and Gynecology, Wilmot Cancer Institute, University of Rochester Medical Center, Rochester, NY USA; 3 Department of Pathology, Microbiology, and Immunology, University of Nebraska Medical Center, Omaha, NE USA; 4 ANGLE North America, Plymouth Meeting, PA USA

**Keywords:** Circulating tumor cells, Parsortix^®^ PC1 system, Metastatic breast cancer, Liquid biopsy

## Abstract

Circulating Tumor Cells (CTCs) may serve as a non-invasive source of tumor material to investigate an individual’s disease in real-time. The Parsortix^®^ PC1 System, the first FDA-cleared medical device for the capture and harvest of CTCs from peripheral blood of metastatic breast cancer (MBC) patients for use in subsequent user-validated downstream analyses, enables the epitope-independent capture of CTCs with diverse phenotypes based on cell size and deformability. The aim of this study was to determine the proportion of MBC patients and self-declared female healthy volunteers (HVs) that had CTCs identified using immunofluorescence (IF) or Wright-Giemsa (WG) staining. Peripheral blood from 76 HVs and 76 MBC patients was processed on Parsortix^®^ PC1 Systems. Harvested cells were cytospun onto a charged slide and immunofluorescently stained for identification of CTCs expressing epithelial markers. The IF slides were subsequently WG-stained and analyzed for CTC identification based on morphological features of malignant cells. All testing was performed by operators blinded to the clinical status of each subject. CTCs were identified on the IF slides in 45.3% (≥ 1) / 24.0% (≥ 5) of the MBC patients (range = 0 – 125, mean = 7) and in 6.9% (≥ 1) / 2.8% (≥ 5) of the HVs (range = 0 – 28, mean = 1). Among the MBC patients with ≥ 1 CTC, 70.6% had only CK + /EpCAM- CTCs, with none having EpCAM + /CK- CTCs. CTC clusters were identified in 56.0% of the CTC-positive patients. On the WG-stained slides, CTCs were identified in 42.9% (≥ 1) / 21.4% (≥ 5) of the MBC patients (range = 0 – 41, mean = 4) and 4.3% (≥ 1) / 2.9% (≥ 5) of the HVs (range = 0 – 14, mean = 0). This study demonstrated the ability of the Parsortix^®^ PC1 System to capture and harvest CTCs from a significantly larger proportion of MBC patients compared to HVs when coupled with both IF and WG cytomorphological assessment. The presence of epithelial cells in subjects without diagnosed disease has been previously described, with their significance being unclear. Interestingly, a high proportion of the identified CTCs did not express EpCAM, highlighting the limitations of using EpCAM-based approaches.

**Supplementary Information:**

The online version contains supplementary material available at 10.1186/s13046-024-03149-x.

## Background

Securing tissue biopsy samples from metastatic tumors, particularly from certain organ sites, is highly invasive and complex. Alternative approaches include collection of tumor material from more easily accessible bodily fluids such as blood or urine to assess various phenotypic and/or genotypic aspects of the tumor’s biology [1, 2]. The minimally invasive collection of blood, also referred to as a liquid biopsy, offers the potential to characterize tumors at genetic, transcriptional and protein levels and allows the opportunity to perform routine, repeated characterizations for monitoring a patients’ disease status and developing effective personalized treatments [3–6].

Circulating Tumor Cells (CTCs) are cells shed by solid tumors that migrate into the blood stream and disseminate. CTCs may extravasate through the endothelial cell layer into different tissues to form metastases in distant organs [7–9]. It is well known that CTCs can be used to predict disease progression and overall survival in patients with metastatic breast cancer (MBC) [10–15] and represent a reliable surrogate marker of treatment response and a potential alternative form of non-invasive monitoring of response to therapy [16, 17]. Deoxyribonucleic acid (DNA), Ribonucleic acid (RNA) and proteins can be obtained from viable CTCs isolated from peripheral blood, offering invaluable insights into the biology of a cancer. Until recently, however, the process of isolating CTCs from blood was very challenging, limiting their routine use in the clinical setting [18–20].

CTCs are rare, representing an extremely small fraction of the cells present in a blood sample. Among the technologies developed to isolate CTCs, the CELLSEARCH® System (Menarini Silicon BioSystems) [21] is Food and Drug Administration (FDA)-cleared for CTC enumeration only. This method involves immune-affinity separation using antibodies against the Epithelial Cell Adhesion Molecule (EpCAM), leading to the selective isolation of a particular CTC phenotype that is likely not completely representative of all the cells being shed from the tumor and can potentially impact outcomes of gene expression analyses [22–25].

ANGLE developed the Parsortix^®^ PC1 System, a semi-automated device capable of capturing and harvesting CTCs from bodily fluids based on cell size and lack of deformability. The isolation/capture mechanism employed is purely physical, rather than epitope-dependent, allowing the system to capture cells with a variety of different phenotypes, such as epithelial or mesenchymal. The system employs a separation cassette (GEN3P6.5) containing a microfluidic structure comprising a series of steps across which cells flow, leading to a smaller critical gap. Most of the common blood cells and components (i.e. red blood cells (RBCs), white blood cells (WBCs), and platelets) pass across the critical gap, while CTC are retained in the separation cassette due to their size and rigidity, together with a small number of residual WBCs [26, 27].

The observational study reported in this manuscript, referred to hereinafter as the ANG-008 study, was designed to demonstrate that the Parsortix^®^ PC1 System could capture and harvest CTCs from the peripheral blood of patients with MBC and that the CTCs harvested by the System could be used for subsequent downstream evaluation with immunofluorescence (IF) and cytology evaluations.

The following objectives defined in the ANG-008 clinical study are detailed in this manuscript:

- Determine the proportion of MBC patients and female healthy volunteers (HVs or controls) that had one or more observable epithelial CTCs (as determined by IF) harvested from their peripheral blood using the Parsortix^®^ PC1 System.
- Determine the proportion of MBC patients and female healthy volunteers (HVs or controls) that had one or more observable CTCs (as determined by cytomorphological review of the IF-stained slides that have been Wright-Giemsa (WG) stained) harvested from their peripheral blood using the Parsortix^®^ PC1 System and compare these results to the IF results.

The data generated from the ANG-008 study was used to support a De Novo request (DEN200062) for re-classification of the Parsortix® PC1 System as a Class II prescription device for use in MBC patients to capture and harvest CTCs for subsequent, user-validated, downstream evaluation, which was granted by the FDA on May 24, 2022 [28].

## Materials and methods

### Ethical conduct of the study

The ANG-008 study was sponsored by ANGLE Europe Ltd (ANGLE) and involved the collection of whole blood samples from healthy women as well as from women with metastatic breast cancer. The study was considered to be exempt from the IDE (investigational device exemption) regulations (21 Code of Federal Regulations (CFR) Part 812.2.c.3) due to the fact that the only procedure required for participation in the study was the collection of blood samples, which is considered to be non-invasive, as well as the fact that none of the results of the research testing were reported back to the subjects and/or the investigators, or used in the diagnosis, treatment and/or care of the subjects.

This study was conducted in a manner consistent with:

- United States (US) standards of Good Clinical Practice (GCP) as defined in US FDA CFR, particularly 21 CFR Part 812 (i.e. Sponsor & Investigator responsibilities), Part 50 (Informed Consent Requirements), Part 54 (Financial Disclosure), Part 56 (IRB Approval) and Part 11 (Electronic Records);
- International GCP standards using the International Conference on Harmonization (ICH) guidelines on GCP;
- Applicable FDA regulations;
- Institutional Review Board(s) (IRB) requirements.

### Sample size calculation

Based on the preliminary data and literature review, the hypothesis that the Parsortix^®^ PC1 System would be able to harvest observable CTCs as identified by IF in ≥ 25% of the MBC patients and in ≤ 3% of the HV control group was used. Assuming an overall study failure rate of ~ 5% (e.g., ineligible subjects, insufficient volume of blood, processing failures, etc.), it was expected that ~ 80 HVs and ~ 80 MBC patients would need to be enrolled to ensure a minimum of 75 HV subjects and 75 MBC patients with evaluable IF results for evaluation of the objective.

- With a sample size of N = 75 evaluable MBC patients, a two-sided 95% confidence interval (CI) for a single proportion, using the large sample normal approximation, will extend a maximum of ± 11.4% from the actual proportion of MBC patients found to have observable CTCs. With a sample size of N = 75 evaluable HVs, a two-sided 95% CI for a single proportion, using the large sample normal approximation, will extend a maximum of ± 3.9% from the actual proportion of HVs with observable CTCs for an expected proportion of ≤ 3%.
- Additionally, with a sample size of 75 evaluable MBC patients and 75 HVs, a two-group continuity corrected chi-square test with a 0.05 two-sided significance level (α) will have ~ 95% power (1—β) to detect a difference between a proportion of > 25% for MBC patients with observable CTCs and a proportion of < 3% for HV subjects with observable CTCs.

### Enrollment and sample collection

All study participants provided informed consent before being enrolled in the study. Each subject was only entered into the study once. All laboratory testing was performed by operators blinded to the clinical status of the participants. A total of 76 female HVs and 85 MBC patients were enrolled between July 2019 and November 2019 at the clinical study site (University of Rochester Medical Center, Rochester, NY).

Inclusion criteria for the MBC patients were as follows:

- Female ≥ 22 years of age;
- Documented evidence of metastatic breast cancer (i.e. primary tumor histopathology of breast cancer and documented evidence of distant sites of metastasis by imaging, biopsy, and/or other means);
- Willing and able to provide informed consent and agree to complete all aspects of the study.

The inclusion criteria for the HV subjects are detailed below. The information obtained from the HVs was ‘self-reported’, as complete medical records were not available at the enrolling site for these control subjects.

- Females ≥ 22 years of age;
- No known fever or active infections at the time of the blood collection;
- No known current diagnosis of acute inflammatory disease or chronic inflammation;
- No known current and/or prior history of malignancy, excluding skin cancers (squamous cell or basal cell);
- Willing and able to provide informed consent and agrees to complete all aspects of the study.

**Fig. 1:**
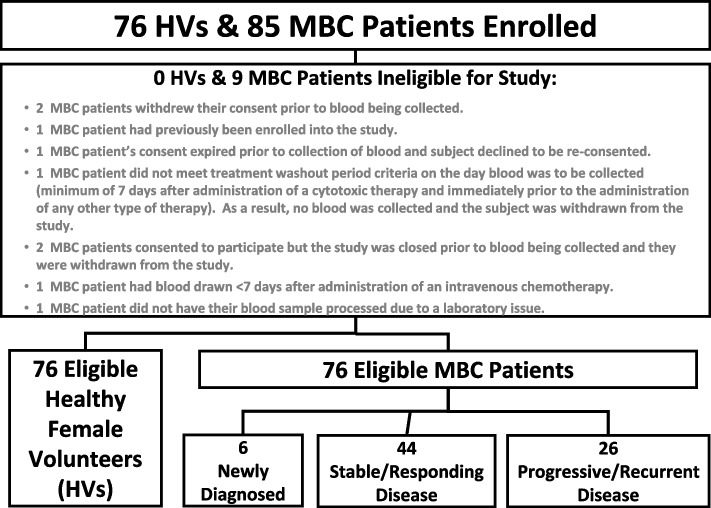
CONSORT Diagram for ANG-008 Study Subject Eligibility. Diagram shows enrolled patients, reasons for ineligibility within the MBC group, and breakdown of MBC patients in newly diagnosed, stable/responding diseases and progressive/recurrent disease groups

None (0%) of the 76 HVs were found to be ineligible. A total of 9 (10.5%) of the 85 MBC patients enrolled were found to be ineligible or not usable for the study, leaving a total of 76 eligible MBC patients (Fig. 1).

Four tubes of blood (one 3 mL K_2_ Ethylenediaminetetraacetic acid (EDTA) vacutainer for CBC with differential and erythrocyte sedimentation rate testing, two 10 mL K_2_EDTA vacutainers for processing on the Parsortix^®^ System, and one 7.5 mL Serum-separating tube (SST) vacutainer for serum chemistry and lipid panel testing) were collected by venipuncture (or, for MBC patients, if applicable, through a venous port) from each HV subject and from each MBC patient, a minimum of seven days after the administration of a cytotoxic therapy (intravenously administered) and immediately prior to the administration of any other type of therapy. For the objectives detailed in this report, an average of 8.6 ± 1.2 mL of blood from one of the 10 mL K_2_EDTA vacutainers was processed on Parsortix^®^ PC1 Systems and the population of cells harvested were deposited onto cytology slides for cytopathological evaluation using IF and WG staining.

A breakdown of the ages, demographics, and clinical information for the eligible HV subjects and MBC patients is provided in Table 1.

**Table 1:** Summary of eligible participants’ clinical characteristics. Table shows demographics in *N*=76 HV subjects and *N*=76 MBC patients who were eligible for the study. The p-values for BSA, BMI and age are for the comparison of the medians for these parameters between the HV subjects and the MBC patient groups and were calculated using a non-parametric chi-squared test for equality of the medians (due to the non-normal distribution of the data), while the p-values for the comparison of the proportions of subjects in the various clinical and demographical groupings between the HV subjects and MBC patient groups were determined using a Fisher’s exact test

**Eligible Participants**	**Healthy Volunteers**	**MBC Patients**	***p*-value**
**# Of Eligible Participants**	**76**	**76**	**---**
**Age (Average ± Standard Deviation (SD) [Median])**	39 ± 12 [36]	64 ± 11 [66]	0.000
**Body Surface Area (BSA) (m**^**2**^**) [Avg ± SD (Median)]**	1.8 ± 0.2 [1.8]	1.8 ± 0.2 [1.8]	1.000
**Body Mass Index (BMI) [Avg ± SD (Median)]**	28.2 ± 6.5 [27.7]	28.6 ± 6.3 [27.1]	0.516
**Age at the time of the blood collection**			
** <30**	15 (19.7%)	0 (0.0%)	0.000
** 30 - 39**	30 (39.5%)	2 (2.6%)	
** 40 - 49**	15 (19.7%)	7 (9.2%)	
** 50 - 59**	11 (14.5%)	13 (17.1%)	
** 60 - 69**	5 (6.6%)	26 (34.2%)	
** ≥70**	0 (0.0%)	28 (36.8%)	
**Age at the time of the blood collection**			
** <65**	74 (97.4%)	37 (48.7%)	0.000
** ≥65**	2 (2.6%)	39 (51.3%)	
**Blood Collection Method**			
** Venipuncture**	76 (100.0%)	32 (42.1%)	0.000
** Port**	0 (0.0%)	44 (57.9%)	
**Menopausal Status**			
** Pre-Menopausal**	56 (73.7%)	7 (9.2%)	0.000
** Post-Menopausal**	17 (22.4%)	66 (86.8%)	
** Unknown**	3 (3.9%)	3 (3.9%)	
**Race**			
** White**	69 (90.8%)	69 (90.8%)	0.013
** Black**	1 (1.3%)	7 (9.2%)	
** Hispanic**	1 (1.3%)	0 (0.0%)	
** Asian**	3 (3.9%)	0 (0.0%)	
** Mixed**	2 (2.6%)	0 (0.0%)	
**Smoking Status**			
** Never Smoked**	49 (64.5%)	35 (46.1%)	0.046
** Previous Smoker**	24 (31.6%)	36 (47.4%)	
** Current Smoker**	2 (2.6%)	5 (6.6%)	
** Unknown**	1 (1.3%)	0 (0.0%)	
**Previous History of Cancer?**			
** Yes**	1 (1.3%)	6 (7.9%)	0.116
** No**	75 (98.7%)	70 (92.1%)	
**Current Dx of Hypertension?**			
** Yes**	4 (5.3%)	42 (55.3%)	0.000
** No**	72 (94.7%)	34 (44.7%)	
**Current Dx of High Cholesterol?**			
** Yes**	3 (3.9%)	18 (23.7%)	0.001
** No**	73 (96.1%)	58 (76.3%)	
**Taking Growth Factors?**			
** Yes**	0 (0.0%)	3 (3.9%)	0.245
** No**	76 (100.0%)	73 (96.1%)	
**Taking Anti-Coagulants?**			
** Yes**	1 (1.3%)	13 (17.1%)	0.001
** No**	75 (98.7%)	63 (82.9%)	
**Taking Anti-Inflammatories?**			
** Yes**	5 (6.6%)	36 (47.4%)	0.000
** No**	71 (93.4%)	40 (52.6%)	
**Taking Pain Medications?**			
** Yes**	3 (3.9%)	49 (64.5%)	0.000
** No**	73 (96.1%)	27 (35.5%)	
**Receiving Cytotoxic Therapies?**			
** Yes**	0 (0.0%)	33 (43.4%)	0.000
** No**	76 (100.0%)	43 (56.6%)	
**ER Status**			
** Positive**	---	66 (86.8%)	
** Negative**	---	9 (11.8%)	
** Unknown**	---	1 (1.3%)	
**PR Status**			
** Positive**	---	56 (73.7%)	
** Negative**	---	19 (25.0%)	
** Unknown**	---	1 (1.3%)	
**HER2-neu Status**			
** Positive**	---	16 (21.1%)	
** Equivocal**	---	7 (9.2%)	
** Negative**	---	45 (59.2%)	
** Unknown**	---	8 (10.5%)	
**MBC Patient Disease Status**			
** Newly Diagnosed**	---	6 (7.9%)	
** Stable / Responding**	---	44 (57.9%)	
** Progressive / Recurrent**	---	26 (34.2%)	
**Sites of Metastasis (more than one may apply)**			
** Abdomen (Abdominal Cavity)**	---	0 (0.0%)	
** Adrenal Gland**	---	0 (0.0%)	
** Ascites**	---	0 (0.0%)	
** Bone**	---	60 (78.9%)	
** Brain**	---	1 (1.3%)	
** Chest Wall**	---	3 (3.9%)	
** Kidney**	---	1 (1.3%)	
** Liver**	---	15 (19.7%)	
** Lung**	---	14 (18.4%)	
** Lymph Nodes**	---	20 (26.3%)	
** Other Site(s)**	---	6 (7.9%)	

The demographics of the MBC patient population was consistent with the demographics of MBC patients described in the literature [29]. Approximately one-third of the MBC patients enrolled had progressive / recurrent metastatic disease (35.7%), 7.9% had newly diagnosed disease, with the largest proportion having stable/responding disease (57.9%). The race distribution is typical of most US based clinical trials, with the majority of patients having a white background. The breast cancer phenotype for most of the MBC patients was Estrogen Receptor (ER) /Progesterone Receptor (PR) positive and Human epidermal growth factor receptor-2 (HER2) negative, with approximately 89% being ER and/or PR positive and 21.1% having HER2 positive breast cancer. Bone was the most prevalent site of metastatic disease (67.1%), followed by the lymph nodes (26.3%), the liver (19.7%) and/or the lungs (18.4%), which are the most common sites of breast cancer metastasis reported in the literature [30, 31]. There was a significant difference observed between the age and menopausal status of the HV subjects compared to the MBC patients, as the majority of the HV subjects were much younger compared to the MBC patients. This also led to significantly lower proportions of HV subjects with comorbidities and those taking medications compared to the MBC patients.

### Blood processing on Parsortix^®^ PC1 instrument

Blood separation was performed at the Targeted Therapeutics Laboratory at the Wilmot Cancer Institute within eight hours from blood draw using Parsortix^®^ PC1 Systems. The Parsortix^®^ PC1 System is a bench top laboratory instrument consisting of inbuilt computer, pneumatic and hydraulic components, and other electronics to control the instrument hardware and behavior. The Parsortix^®^ PC1’s proprietary application software runs a series of encrypted Protocol Files (Clean, Prime, Separate, and Harvest) to control the instrument fluidic and hydraulic components. The instrument utilizes a single use, non-sterile Parsortix^®^ GEN3 Cell separation cassette, containing precision molded separation structures with ‘step’ configurations. Whole blood flows along a series of channels under controlled and constant pressure conditions (99 mbar) to enable separation. The channel height progressively decreases at each step toward the final ‘critical gap’. As a result, in the case of blood, cells are captured in the critical gap based on their size and resistance to compression. The looped cassette layout is designed to maximize the width of separating steps, which is a key factor affecting separation capability and capture capacity, providing fluid paths with minimal resistance to liquid flow. The cassette layout is intentionally omni-directional such that during a separation, the sample always flows across the step structures and then through the critical gap. To harvest cells captured in the cassette, this flow is intentionally reversed to release the cells from the critical gap and step structures and flush them out of the cassette into another receptacle using a small volume of buffer (~ 210 µL).

### Cytology slide preparation

The Targeted Therapeutics Laboratory prepared the cytology slides for shipment to ANGLE Guildford central laboratory where the IF evaluations were performed. Following separation and enrichment, captured cells were harvested into a 1.5 mL microfuge tube containing 60 μL of fetal bovine serum (FBS). The harvested cells and FBS mixture was pipetted into a Cytospin® 4 Cytofunnel™ assembly (Thermo Fisher Scientific) containing a positively charged glass Shandon™ Single Cytoslides™ (Thermo Fisher Scientific). The slide assembly was cytocentrifuged at 800 rpm for 3 min on low acceleration, and the slide was removed from the assembly and allowed to air-dry at room temperature for 1 min. The air-dried slide was then submersed in ice-cold 100% acetone for 5 min at -20 °C and allowed to air-dry at room temperature for 30 min. The fixed slides were stored refrigerated (at + 2–8 °C) and shipped weekly to the ANGLE Guildford central laboratory for staining and evaluation.

### Immunofluorescence staining and imaging

The development of the IF assay used in this study is described in Additional Files 1, 2, 3, 4, 5 and 6. The procedure is summarized below.

Slides were kept refrigerated until IF staining was performed. Before staining, each slide was re-hydrated with 1 × Phosphate Buffered Saline (PBS) for 60 min. After re-hydration, slides were blocked with 2.5% Normal Horse Serum (S-2012 Vector Labs) and stained with an antibody mixture against surface blood lineage markers (CD45-Allophycocyanin (APC), CD16-APC, CD11b-APC and CD61-APC diluted in 1 × PBS) followed by another antibody mixture against intracellular markers (Cytokeratin (CK) 8-Alexa Fluor 488 (AF488), CK18-AF488, CK19-AF488, EpCAM-Alexa Fluor 555 (AF555), and 4′,6-diamidino-2-phenylindole (DAPI) diluted in Inside Perm (Miltenyi Biotec)). Slides were mounted with 50 µL of 1 × PBS, a 25 mm × 25 mm glass coverslip and fixogum.

Slides were examined using a Leica LAS X fluorescence microscope or a BioView Allegro Plus imaging system, and the cells of interest (i.e. CTCs) were classified based on their staining patterns as follows: 1) EpCAM + , CK + , CD-, DAPI + ; 2) EpCAM + , CK-, CD-, DAPI + ; and 3) EpCAM-, CK + , CD-, DAPI + .

### Wright-Giemsa staining and cytological evaluation

Upon completion of the IF evaluation, the coverslips were removed from each of the slides, and the slides were air dried and stored at room temperature until shipment to the Department of Hematopathology, Division of Pathology and Laboratory Medicine, at MD Anderson Cancer Center for WG staining and cytopathological evaluation. The slides underwent Richard-Allen Scientific WG staining on an automated stainer and examination by a qualified pathologist with expertise in blood evaluation and cytopathology (Dr. Joseph Khoury, JDK) using light microscopy. CTCs were identified and enumerated using conventional cytomorphologic criteria for malignancy, which included: size larger than peripheral WBCs, moderate to abundant cytoplasm, cytoplasmic vacuoles (micro or macro), irregular nuclear contours, nuclear hyperchromasia and prominent nucleoli. A CTC cluster was defined as 3 or more cohesive cells [32].

## Results

### Immunofluorescence samples evaluation

**Fig. 2:**
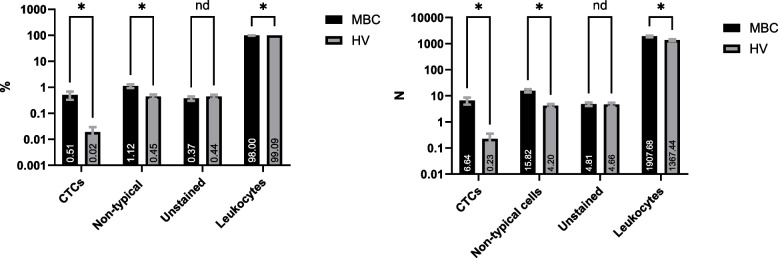
Breakdown of cell populations present in the harvests of HV and MBC subjects. Histograms show mean ± Standard Error of the Mean (SEM) of the (**A**) percentage and (**B**) absolute number of cells in each population (Multiple Mann Whitney test, *P < 0.05, nd = discovery non-significant). Mean number is noted on each column

Four of the 76 eligible HV subjects and one of the 76 eligible MBC patients had non-evaluable IF samples, leaving a total of 72 HV subjects and 75 MBC patients with evaluable IF stained slides. On the evaluable IF samples, four nucleated cells’ populations were identified: leukocytes ("Leukocytes" sect.), CTCs ("Circulating Tumor Cells" sect.), a cell population defined as other non-typical circulating cells ("Other Non-Typical Circulating Cells" sect.) and nucleated cells that remained unstained, i.e. negative for the epithelial, mesenchymal and CD markers included in the IF panel ("Unstained Cells" sect.). The breakdown of each cell population is shown in Fig. 2.

#### Leukocytes

**Fig. 3:**
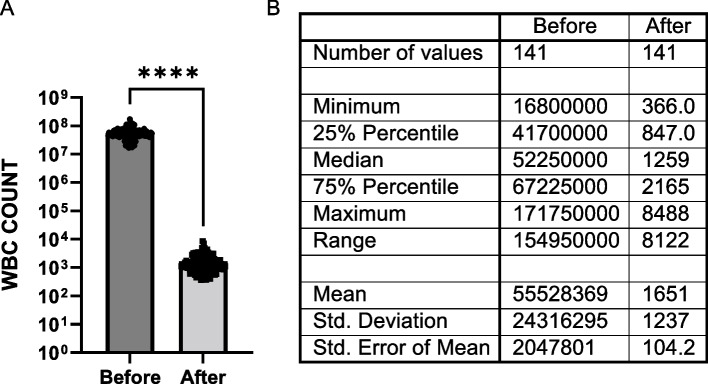
Performance of Parsortix^®^ PC1 System in eliminating leukocytes from whole blood for CTCs enrichment. **A** Histogram shows mean ± SEM of the number of WBCs present in the blood samples before enrichment vs the number of harvested WBCs (*p* ≥ 0.0001, Paired t-test) after enrichment for each donor. Graph includes all MBC and HV subjects with initial evaluable CBC count; **B** Table shows descriptive statistics

All cells that were nucleated, negative for CK/EpCAM, positive for CD markers and smaller than 20 µm in diameter were considered leukocytes. Since all four CD markers (CD45, CD16, CD11b and CD61) were combined and detected under one fluorescence channel, it was not possible to specify the subtypes of cells in this population. While leukocytes represented 98% of the nucleated cells identified on the MBC patients’ slides (mean: 1,908 cells per slide) and 99% of the cells identified on the HVs’ slides (mean: 1,367 cells per slide), it is important to note that the Parsortix^®^ PC1 System was highly efficient in enriching CTCs and eliminating the blood cells component from the starting blood samples, with a purity of > 99%, calculated as a percentage of the mean WBC difference before and after processing over the mean WBC count before processing: (Fig. 3).

#### Circulating tumor cells

**Fig. 4:**
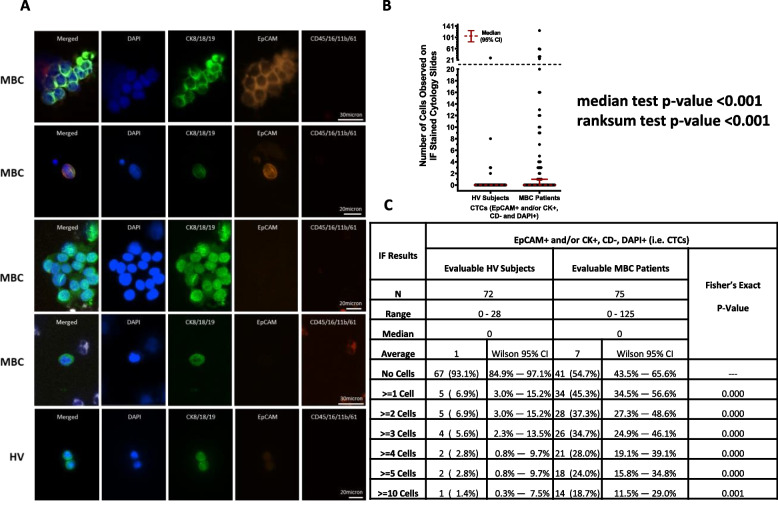
IF evaluation results. **A** Representative images of CK+, EpCAM +/- CTCs and CTCs clusters identified in MBC patients and HV subjects (CKs-AF488) in green, EpCAM-AF555 in orange, Blood lineage markers (APC) in red, Nucleus (DAPI) in blue). **B** Dot plot shows median ± 95% CI of the number of CTCs identified in each MBC and HV donor by IF. A statistically higher number of CTCs was found in MBC patients compared to HVs (p≥0.001, Median test). **C** Table shows number of donors included in each cohort (N), range, median and average number of CTCs identified within each cohort, and, using CTC thresholds of 0, ≥1, ≥2, ≥3, ≥4, ≥5 and ≥10 CTCs identified, the number and percentage of donors within each CTC category along with Wilson 95% CI’s for each proportion. The Fisher’s exact test *p*-values shown are for the comparison of the proportions of HV subjects and MBC patients with less than vs. greater than or equal to varying numbers of CTCs observed on the IF slides, and in each instance, a significantly higher proportion of MBC patients were CTC positive compared to the HV subjects

Only DAPI + cells that were also CD- were further evaluated for expression of CKs and/or EpCAM. CTCs were defined as cells that were DAPI + , CD- and CK + and/or EpCAM + . The results of the IF evaluation are summarized in Fig. 4. Out of the 75 MBC patients with evaluable IF results, 41 (54.7%, Wilson 95% CI = 43.5% – 65.6%) had no cells classified as being epithelial CTCs, whereas 34 (45.3%, Wilson 95% CI = 34.5% – 56.6%) had one or more cells observed on their IF slides that were DAPI + , EpCAM + and/or CK + , and CD-, while 18 (24.0%, Wilson 95% CI = 15.8% – 34.8%) had five or more cells observed on their IF slides that were DAPI + , EpCAM + and/or CK + , and CD-. In the 34 MBC patients with one or more epithelial CTCs observed, 70.6% had only CK + , EpCAM- cells while the remaining 29.4% had ≥ 1 CK + , EpCAM + cell. No EpCAM + , CK- CTCs were identified in MBC patients. Among the CTC-positive MBC patients, clusters of CTCs, defined as two or more individual CTCs co-aggregating with or without the presence of leukocytes, were identified in 19 MBC patients (56%), with a range of 1-8 clusters per patient and a range of 2-44 CTCs per cluster. Of the 19 patients with CTCs clusters, 11 (58%) had at least one heterogeneous cluster defined as an aggregation of ≥ 2 CTCs and ≥ 1 leukocyte.

In the 72 HV subjects with evaluable IF results, 67 (93.1%, Wilson 95% CI = 84.9% – 97.1%) had no cells classified as being CTCs whereas 5 (6.9%, Wilson 95% CI = 3.5% – 15.2%) had one or more cells observed on their IF slides that were DAPI + , EpCAM + and/or CK + , and CD-. One of the five CTC-positive HVs had only CK + , EpCAM + cells, three had only CK + , EPCAM- cells, while the remaining donor had a combination of both. Among the five CTC-positive HV subjects, one had 28 CTCs (this subject was identified as being pregnant at the time of their blood collection), one had 8 CTCs and the remaining three had ≤ 5 CTCs on their IF stained slides.

Taken together, as shown in Fig. 4, a significantly higher proportion of MBC patients were CTCs positive compared to the HV subjects using any cut off for CTCs from ≥ 1 to ≥ 10.

Table 2 below summarizes the proportions of HV subjects and MBC patients with cells observed on their IF stained cytology slides that were classified as CTCs within various demographical and clinical characteristic subgroupings. Although the number of MBC patients in this study was relatively small, it is interesting to note that:

**Table 2:** Epithelial CTC Prevalence Rates from IF Stained Cytology Slides by Various Demographics and Clinical Characteristics. The Fisher’s exact test p-values shown (when applicable) are for the comparison of the proportions of either HV subjects or MBC patients with and without one or more epithelial CTCs or five or more epithelial CTCs between the various clinical and demographical groupings

Parameter and Categories	Eligible Subjects with Evaluable IF Stained Cytology Slides					
	**All HV Subjects**			**All MBC Patients**		
	**N**	** > = 1 CTC**	** > = 5 CTC**	**N**	** > = 1 CTC**	** > = 5 CTC**
**All Patients**	**72**	**5 (6.9%)**	**2 (2.8%)**	**75**	**34 (45.3%)**	**18 (24.0%)**
**Age at the time of the blood collection**						
** < 30**	14	1 (7.1%)	0 (0.0%)	0	0 (0.0%)	0 (0.0%)
** 30—39**	30	1 (3.3%)	0 (0.0%)	2	2 (100.0%)	2 (100.0%)
** 40—49**	14	2 (14.3%)	1 (7.1%)	7	4 (57.1%)	0 (0.0%)
** 50—59**	10	1 (10.0%)	1 (10.0%)	13	7 (53.8%)	2 (15.4%)
** 60—69**	4	0 (0.0%)	0 (0.0%)	25	12 (48.0%)	9 (36.0%)
** > = 70**	0	0 (0.0%)	0 (0.0%)	28	9 (32.1%)	5 (17.9%)
** Fisher’s Exact Test** ***p*** **-value**		0.610	0.307		0.288	0.034
**Blood Collection Method**						
** Venipuncture**	72	5 (6.9%)	2 (2.8%)	32	8 (25.0%)	3 (9.4%)
** Port**	0	0 (0.0%)	0 (0.0%)	43	26 (60.5%)	15 (34.9%)
** Fisher’s Exact Test** ***p*** **-value**		–-	–-		0.003	0.014
**Volume of Blood Processed**						
** ≤ 8.0 mL**	21	0 (0.0%)	0 (0.0%)	17	4 (23.5%)	3 (17.6%)
** 8.5—9.0 mL**	30	3 (10.0%)	1 (3.3%)	23	13 (56.5%)	8 (34.8%)
** 9.5 mL**	15	2 (13.3%)	1 (6.7%)	23	12 (52.2%)	5 (21.7%)
** ≥ 10.0 mL**	6	0 (0.0%)	0 (0.0%)	12	5 (41.7%)	2 (16.7%)
** Fisher’s Exact Test** ***p*** **-value**		0.334	0.754		0.180	0.628
**Menopausal Status**						
** Pre-Menopausal**	54	3 (5.6%)	1 (1.9%)	7	4 (57.1%)	2 (28.6%)
** Post-Menopausal**	15	1 (6.7%)	1 (6.7%)	65	29 (44.6%)	16 (24.6%)
** Unknown**	3	1 (33.3%)	0 (0.0%)	3	1 (33.3%)	0 (0.0%)
** Fisher’s Exact Test** ***p*** **-value**		0.249	0.440		0.874	1.000
**Race**						
** White**	65	3 (4.6%)	1 (1.5%)	68	30 (44.1%)	17 (25.0%)
** Black**	1	0 (0.0%)	0 (0.0%)	7	4 (57.1%)	1 (14.3%)
** Other**	6	2 (33.3%)	1 (16.7%)	0	0 (0.0%)	0 (0.0%)
** Fisher’s Exact Test** ***p*** **-value**		0.071	0.186		0.695	1.000
**Smoking Status**						
** Never Smoked**	46	2 (4.3%)	0 (0.0%)	35	12 (34.3%)	6 (17.1%)
** Previous Smoker**	23	3 (13.0%)	2 (8.7%)	35	18 (51.4%)	10 (28.6%)
** Current Smoker**	2	0 (0.0%)	0 (0.0%)	5	4 (80.0%)	2 (40.0%)
** Unknown**	1	0 (0.0%)	0 (0.0%)	0	0 (0.0%)	0 (0.0%)
** Fisher’s Exact Test** ***p*** **-value**		0.417	0.158		0.113	0.326
**Previous History of Cancer?**						
** Yes**	1	0 (0.0%)	0 (0.0%)	6	4 (66.7%)	2 (33.3%)
** No**	71	5 (7.0%)	2 (2.8%)	69	30 (43.5%)	16 (23.2%)
** Fisher’s Exact Test** ***p*** **-value**		1.000	1.000		0.401	0.626
**Current Diagnosis of Hypertension?**						
** Yes**	4	0 (0.0%)	0 (0.0%)	41	14 (34.1%)	8 (19.5%)
** No**	68	5 (7.4%)	2 (2.9%)	34	20 (58.8%)	10 (29.4%)
** Fisher’s Exact Test** ***p*** **-value**		1.000	1.000		0.039	0.417
**Current Diagnosis of High Cholesterol?**						
** Yes**	2	0 (0.0%)	0 (0.0%)	17	9 (52.9%)	7 (41.2%)
** No**	70	5 (7.1%)	2 (2.9%)	58	25 (43.1%)	11 (19%)
** Fisher’s Exact Test** ***p*** **-value**		1.000	1.000		0.582	0.103
**Current Taking Growth Factors?**						
** Yes**	0	0 (0.0%)	0 (0.0%)	3	1 (33.3%)	0 (0.0%)
** No**	72	5 (6.9%)	2 (2.8%)	72	33 (45.8%)	18 (25.0%)
** Fisher’s Exact Test** ***p*** **-value**		–-	–-		1.000	1.000
**Current Taking Anti-Coagulants?**						
** Yes**	1	1 (100.0%)	1 (100.0%)	13	6 (46.2%)	2 (15.4%)
** No**	71	4 (5.6%)	1 (1.4%)	62	28 (45.2%)	16 (25.8%)
** Fisher’s Exact Test** ***p*** **-value**		0.069	0.028		1.000	0.722
**Current Taking Anti-Inflammatories?**						
** Yes**	4	1 (25.0%)	1 (25.0%)	35	17 (48.6%)	9 (25.7%)
** No**	68	4 (5.9%)	1 (1.5%)	40	17 (42.5%)	9 (22.5%)
** Fisher’s Exact Test** ***p*** **-value**		0.255	0.109		0.647	0.791
**Current Taking Pain Medications?**						
** Yes**	3	0 (0.0%)	0 (0.0%)	48	27 (56.3%)	15 (31.3%)
** No**	69	5 (7.2%)	2 (2.9%)	27	7 (25.9%)	3 (11.1%)
** Fisher’s Exact Test** ***p*** **-value**		1.000	1.000		0.016	0.089
**Current Taking Cytotoxic Therapies?**						
** Yes**	0	0 (0.0%)	0 (0.0%)	32	17 (53.1%)	7 (21.9%)
** No**	72	5 (6.9%)	2 (2.8%)	43	17 (39.5%)	11 (25.6%)
** Fisher’s Exact Test** ***p*** **-value**		–-	–-		0.348	0.789
**Breast Cancer ER Status**						
** Positive**	0	0 (0.0%)	0 (0.0%)	65	29 (44.6%)	13 (20.0%
** Negative**	0	0 (0.0%)	0 (0.0%)	9	4 (44.4%)	4 (44.4%)
** Unknown**	0	0 (0.0%)	0 (0.0%)	1	1 (100.0%)	1 (100.0%)
** Fisher’s Exact Test** ***p*** **-value**		–-	–-		0.849	0.073
**Breast Cancer PR Status**						
** Positive**	0	0 (0.0%)	0 (0.0%)	55	26 (47.3%)	10 (18.2%)
** Negative**	0	0 (0.0%)	0 (0.0%)	19	7 (36.8%)	7 (36.8%)
** Unknown**	0	0 (0.0%)	0 (0.0%)	1	1 (100.0%)	1 (100.0%)
** Fisher’s Exact Test** ***p*** **-value**		–-	–-		0.427	0.051
**Breast Cancer HER2-neu Status**						
** Positive**	0	0 (0.0%)	0 (0.0%)	16	8 (50.0%)	4 (25.0%)
** Equivocal**	0	0 (0.0%)	0 (0.0%)	7	4 (57.1%)	4 (57.1%)
** Negative**	0	0 (0.0%)	0 (0.0%)	44	19 (43.2%)	9 (20.5%)
** Unknown**	0	0 (0.0%)	0 (0.0%)	8	3 (37.5%)	1 (12.5%)
** Fisher’s Exact Test** ***p*** **-value**		–-	–-		0.880	0.194
**MBC Patient Disease Status**						
** Newly Diagnosed**	0	0 (0.0%)	0 (0.0%)	6	2 (33.3%)	2 (33.3%)
** Stable / Responding**	0	0 (0.0%)	0 (0.0%)	44	18 (40.9%)	10 (22.7%)
** Progressive / Recurrent**	0	0 (0.0%)	0 (0.0%)	25	14 (56.0%)	6 (24.0%)
** Fisher’s Exact Test *p*-value**		–-	–-		0.453	0.770

- A significantly increased proportion of MBC patients had one or more cells classified as epithelial CTCs in port collected blood samples compared to venous collected blood samples (≥ 1 CTC: 60.5% vs. 25.0%, respectively, *p*-value = 0.003; ≥ 5 CTC: 34.9% vs. 9.4%, respectively, *p*-value = 0.014).
- A significantly increased proportion of MBC patients taking pain medications had one or more cells classified as epithelial CTCs compared to those not taking pain medications (≥ 1 CTC: 56.3% vs. 25.9%, respectively, *p*-value = 0.016), however this observation was not statistically significant when using a CTC positivity cut off of ≥ 5 CTCs (*p*-value = 0.089).

#### Other non-typical circulating cells

**Fig. 5:**
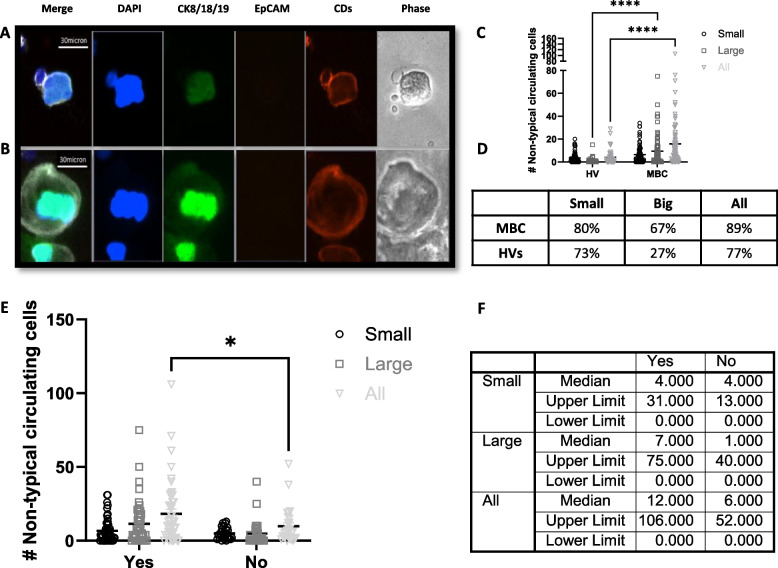
Non-typical circulating cells. **A** Representative image of a small non-typical circulating cell. **B** Representative image of a large non-typical circulating cell. Images were taken using a BioView Allegro Plus imaging system with a 10 × objective lens (CKs-AF488 in green, EpCAM-AF555 in orange, Blood lineage markers-APC in red/white in the merge image, nucleus-DAPI in blue). **C** Dot plot showing mean ± SEM of the number of non-typical circulating cells in each category in the harvest of 72 HV donors and 75 MBC (Two-Way ANOVA followed by Sidak’s multiple comparison test, ****P ≤ 0.0001). **D** Table shows the percentage donors with ≥ 1 non-typical blood cell. **E** Dot plot showing mean ± SEM of the number of non-typical circulating cells of each type in the harvest of 75 MBC (Two-Way ANOVA followed by Sidak’s multiple comparison test, *P ≤ 0.05) divided in patients receiving or not cytotoxic therapy. **F** Table shows median and range numbers of graph **E**

A population of large DAPI + , CK + , CD ± cells with characteristic morphology was identified and classified as “other non-typical circulating cells”. When negative for CD markers, these cells were distinguished from CTCs based on their distinct morphology and CK staining pattern. Non-typical circulating cells accounted on average for 0.45% and 1.12% of all harvested cells in the HV and MBC patient samples, respectively. Two cell populations were identified in this group based on their distinct morphology (Fig. 5):

- “Small” non-typical circulating cells: these cells presented with a low epithelial signal (CK +, EpCAM-negative) and were either positive (~ 80%) or negative (~ 20%) for blood lineage markers. The average diameter was ~ 30 µm, with a large nucleus and small cytoplasmic area, and co-localization between the epithelial and nuclear signals (Fig. 5.A). These cells were found in both HVs and MBC patients at a similar rate. Small non-typical circulating cells accounted for 85% and 53% of all non-typical circulating cells found in the HVs and MBC patient samples in this study, respectively (Fig. 5C).
- “Large” non-typical circulating cells: these cells presented with a low positivity for epithelial markers (CK + , EpCAM-) and were positive for blood lineage markers. These cells were between ~ 40 µm and ~ 80 µm in diameter and usually had a large nucleus and a large cytoplasm (Fig. 5.B). They made up 15% and 47% of all non-typical circulating cells found in the HVs and MBC patient samples in this study, respectively. They were found in both HV and MBC patient samples, but in statistically higher numbers in MBC patient samples (*p* ≤ 0.0001). Additionally, the percentage of donors presenting with large non-typical circulating cells was 2.5-fold higher in MBC patients (67%) compared to HV subjects (27%) (Fig. 5C-D).

Additionally, a statistically significantly higher number of non-typical circulating cells (small and large) were present in MBC patients receiving cytotoxic therapy (Fig. 5E-F) and a weak positive correlation between the number of megakaryocytes and the number of CTCs (Spearman’s r = 0.28; *p* = 0.01) was observed.

#### Unstained cells

A small number of cells were found in both HV and MBC subjects that were nucleated and not stained by any of the epithelial or leukocyte markers used in this study. They represented 0.37% of all cells in MBC samples (mean = 5 cells per donor) and 0.44% of all cells in HV samples (mean = 5 cells per donor), with no statistically significant difference between HVs and MBCs (Fig. 2). In both HV and MBC samples, 91.6% of these cells (0.34%—0.41% of all cells) were smaller than 20 µm in diameter. Based on the size, it is reasonable to assume that these cells are likely to be leukocytes not expressing any of the protein markers used in this study. In both HV and MBC samples, 8.4% of the unstained cells (around 0.03% of all cells) were larger than 40 µm. Morphologically, these cells looked similar to the non-typical circulating cells population.

### Wright-Giemsa samples evaluation

Seven of the 76 eligible HV subjects and six of the 76 eligible MBC patients had non-evaluable WG samples, leaving a total of 69 HV subjects and 70 MBC patients being evaluable for the WG evaluation.

The purpose of the WG evaluation was to determine if the IF-stained slides containing the cells harvested by the Parsortix^®^ PC1 System could be re-stained using WG reagents and evaluated by a pathologist for the identification of malignant cells (CTCs) and to determine the proportions of MBC patients and HV subjects with one or more malignant cells (CTCs) harvested from their peripheral blood using the Parsortix^®^ PC1 System as determined by a pathologist.

**Fig. 6:**
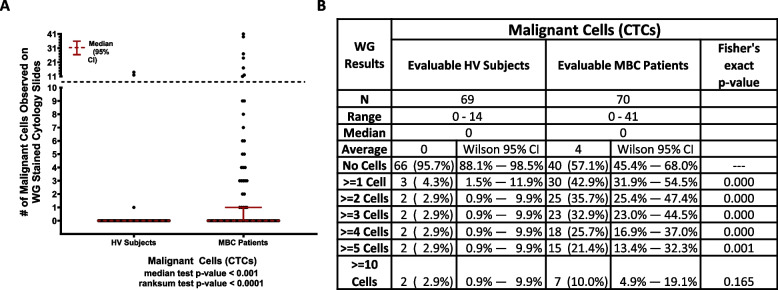
WG evaluation results. **A** Dot plot shows median ± 95% CI of the number of malignant cells (CTCs) identified in each MBC and HV donor by WG. A statistically higher number of CTCs were found in MBC patients compared to HVs (p ≥ 0.001, Median test). **B** Table shows number of donors included in each cohort (N), range, median and average number of CTCs identified within each cohort, and, using CTC thresholds of 0, ≥ 1, ≥ 2, ≥ 3, ≥ 4, ≥ 5 and ≥ 10 CTCs identified, the number and percentage of donors within each CTC category along with Wilson 95% CI’s for each proportion. The Fisher’s exact test p-values shown are for the comparison of the proportions of HV subjects and MBC patients with less than vs. greater than or equal to varying numbers of CTCs observed on the WG-slides, and in each instance up to ≥ 5 CTCs, a significantly higher proportion of MBC patients were CTCs positive compared to the HV subjects

The results of the WG evaluation are summarized in Fig. 6. It was noted by the pathologist during the review of the WG-stained IF cytology slides that there was significant cellular damage observed on the majority of the slides, particularly in the WBCs and RBCs. In the 70 MBC patients with evaluable results from the review of their WG-stained slide, 40 (57.1%, Wilson 95% CI = 45.4% – 68.0%) had no cells classified as being CTCs whereas 30 (42.9%, Wilson 95% CI = 31.9% – 54.5%) had one or more cells observed that were classified as malignant, including 15 (21.4%, Wilson 95% CI = 13.4% – 32.3%) with five or more cells observed that were classified as malignant. In the 40 MBC patients with no malignant cells identified on their WG-stained slides, 14 (35%) had one or more cells classified as CTCs by IF analysis. In the 30 MBC patients with malignant cells identified on their WG-stained slides, 16 (53%) had one or more cells classified as CTCs by IF analysis. In the 69 HV subjects with evaluable results from the review of their WG-stained IF cytology slides, 66 (95.7%, Wilson 95% CI = 88.1% – 98.5%) had no cells classified as being malignant whereas 3 (4.3%, Wilson 95% CI = 1.5% – 11.9%) had one or more cells observed that were classified by the pathologist as being malignant and 2 (2.9%, Wilson 95% CI = 0.9% – 9.9%) had five or more cells observed that were classified as malignant. In the 66 HV subjects with no malignant cells identified by WG analysis, 4 (6%) had one or more cells classified as CTCs by IF analysis. Of the 3 HV subjects with malignant cells identified by WG analysis, none of them had any cells classified as CTCs by IF analysis.

Taken together, as shown in Fig. 6, a significantly higher proportion of MBC patients were CTCs positive compared to the HV subjects using any cut off for CTCs from ≥ 1 to ≥ 5.

Table 3 below summarizes the proportions of HV subjects and MBC patients with cells observed on their WG-stained IF cytology slides that were classified as CTCs within various demographical and clinical characteristic subgroupings. Similar to what was observed in the by IF analysis, a significantly higher proportion of MBC patients with one or more cells classified as malignant was observed in the port collected blood samples compared to the venous blood samples (≥ 1 CTC: 66.7% vs. 12.9%, respectively, *p*-value < 0.001; ≥ 5 CTC: 38.5% vs. 0.0%, respectively, *p*-value < 0.001). It was also found that a significantly greater proportion of MBC patients who reported being on a cytotoxic therapy had one or more cells classified as malignant compared to those not on a cytotoxic therapy (≥ 1 CTC: 63.3% vs. 27.5%, respectively, *p*-value = 0.004; ≥ 5 CTC: 40.0% vs. 7.5%, respectively, *p*-value = 0.002).

**Table 3:** CTC Prevalence Rates from WG-Stained Cytology Slides by Various Demographics and Clinical Characteristics. The Fisher’s exact test p-values shown (when applicable) are for the comparison of the proportions of either HV subjects or MBC patients with and without one or more CTCs or five or more CTCs between the various clinical and demographical groupings

Parameter and Categories	Eligible Subjects with Evaluable WG-Stained Slides					
	**All HV Subjects**			**All MBC Patients**		
	**N**	** > = 1 CTC**	** > = 5 CTC**	**N**	** > = 1 CTC**	** > = 5 CTC**
**All Patients**	**69**	**3 (4.3%)**	**2 (2.9%)**	**70**	**30 (42.9%)**	**15 (21.4%)**
**Age at the time of the blood collection**						
** < 30**	14	1 (7.1%)	1 ( 7.1%)	0	0 ( 0.0%)	0 (0.0%)
** 30—39**	30	1 (3.3%)	1 (3.3%)	2	1 (50.0%)	1 (50.0%)
** 40—49**	13	0 (0.0%)	0 ( 0.0%)	7	6 (85.7%)	3 (42.9%)
** 50—59**	8	1 (12.5%)	0 (0.0%)	12	2 (16.7%)	0 (0.0%)
** 60—69**	4	0 (0.0%)	0 ( 0.0%)	23	13 (56.5%)	6 (26.1%)
** > = 70**	0	0 (0.0%)	0 ( 0.0%)	26	8 (30.8%)	5 (19.2%)
** Fisher’s Exact Test** ***p*-value**		0.528	0.815		0.011	0.097
**Blood Collection Method**						
** Venipuncture**	69	3 (4.3%)	2 ( 2.9%)	31	4 (12.9%)	0 ( 0.0%)
** Port**	0	0 (0.0%)	0 ( 0.0%)	39	26 (66.7%)	15 (38.5%)
** Fisher’s Exact Test** ***p*-value**		–-	–-		0.000	0.000
**Volume of Blood Processed**						
** ≤ 8.0 mL**	19	1 (5.3%)	0 ( 0.0%)	16	4 (25.0%)	1 ( 6.3%)
** 8.5—9.0 mL**	30	2 (6.7%)	2 ( 6.7%)	21	9 (42.9%)	2 ( 9.5%)
** 9.5 mL**	14	0 (0.0%)	0 ( 0.0%)	21	13 (61.9%)	9 (42.9%)
** ≥ 10.0 mL**	6	0 (0.0%)	0 ( 0.0%)	12	4 (33.3%)	3 (25.0%)
** Fisher’s Exact Test** ***p*-value**		1.000	0.757		0.134	0.023
**Menopausal Status**						
** Pre-Menopausal**	54	2 (3.7%)	1 ( 1.9%)	7	4 (57.1%)	3 (42.9%)
** Post-Menopausal**	12	0 (0.0%)	0 ( 0.0%)	60	26 (43.3%)	12 (20.0%)
** Unknown**	3	1 (33.3%)	1 (33.3%)	3	0 ( 0.0%)	0 ( 0.0%)
** Fisher’s Exact Test** *p***-value**		0.199	0.114		0.367	0.263
**Race**						
** White**	62	3 (4.8%)	2 ( 3.2%)	64	26 (40.6%)	12 (18.8%)
** Black**	1	0 (0.0%)	0 ( 0.0%)	6	4 (66.7%)	3 (50.0%)
** Other**	6	0 (0.0%)	0 ( 0.0%)	0	0 ( 0.0%)	0 ( 0.0%)
** Fisher’s Exact Test** ***p*-value**		1.000	1.000		0.391	0.108
**Smoking Status**						
** Never Smoked**	45	2 (4.4%)	2 ( 4.4%)	34	14 (41.2%)	6 (17.6%)
** Previous Smoker**	21	1 (4.8%)	0 ( 0.0%)	31	13 (41.9%)	9 (29.0%)
** Current Smoker**	2	0 (0.0%)	0 ( 0.0%)	5	3 (60.0%)	0 ( 0.0%)
** Unknown**	1	0 (0.0%)	0 ( 0.0%)	0	0 ( 0.0%)	0 ( 0.0%)
** Fisher’s Exact Test** ***p*-value**		1.000	1.000		0.808	0.310
**Previous History of Cancer?**						
** Yes**	1	0 (0.0%)	0 ( 0.0%)	4	1 (25.0%)	0 ( 0.0%)
** No**	68	3 (4.4%)	2 ( 2.9%)	66	29 (43.9%)	15 (22.7%)
** Fisher’s Exact Test** ***p*-value**		1.000	1.000		0.630	0.571
**Current Diagnosis of Hypertension?**						
** Yes**	4	0 ( 0.0%)	0 ( 0.0%)	38	20 (52.6%)	8 (21.1%)
** No**	65	3 ( 4.6%)	2 ( 3.1%)	32	10 (31.3%)	7 (21.9%)
** Fisher’s Exact Test** ***p*-value**		1.000	1.000		0.092	1.000
**Current Diagnosis of High Cholesterol?**						
** Yes**	2	0 ( 0.0%)	0 ( 0.0%)	15	7 (46.7%)	3 (20.0%)
** No**	67	3 ( 4.5%)	2 ( 3%)	55	23 (41.8%)	12 (21.8%)
** Fisher’s Exact Test** ***p*-value**		1.000	1.000		0.775	1.000
**Current Taking Growth Factors?**						
** Yes**	0	0 ( 0.0%)	0 ( 0.0%)	2	2 (100.0%)	1 (50.0%)
** No**	69	3 ( 4.3%)	2 ( 2.9%)	68	28 (41.2%)	14 (20.6%)
** Fisher’s Exact Test** *p***-value**		–-	–-		0.180	0.385
**Current Taking Anti-Coagulants?**						
** Yes**	0	0 ( 0.0%)	0 ( 0.0%)	11	5 (45.5%)	4 (36.4%)
** No**	69	3 ( 4.3%)	2 ( 2.9%)	59	25 (42.4%)	11 (18.6%)
** Fisher’s Exact Test** ***p*-value**		–-	–-		1.000	0.233
**Current Taking Anti-Inflammatories?**						
** Yes**	3	0 ( 0.0%)	0 ( 0.0%)	32	11 (34.4%)	6 (18.8%)
** No**	66	3 ( 4.5%)	2 ( 3.0%)	38	19 (50.0%)	9 (23.7%)
** Fisher’s Exact Test** ***p*-value**		1.000	1.000		0.230	0.772
**Current Taking Pain Medications?**						
** Yes**	3	0 ( 0.0%)	0 ( 0.0%)	46	20 (43.5%)	10 (21.7%)
** No**	66	3 ( 4.5%)	2 ( 3.0%)	24	10 (41.7%)	5 (20.8%)
** Fisher’s Exact Test** ***p*-value**		1.000	1.000		1.000	1.000
**Current Taking Cytotoxic Therapies?**						
** Yes**	0	0 ( 0.0%)	0 ( 0.0%)	30	19 (63.3%)	12 (40.0%)
** No**	69	3 ( 4.3%)	2 ( 2.9%)	40	11 (27.5%)	3 ( 7.5%)
** Fisher’s Exact Test** ***p*-value**		–-	–-		0.004	0.002
**Breast Cancer ER Status**						
** Positive**	0	0 ( 0.0%)	0 ( 0.0%)	62	24 (38.7%)	12 (19.4%)
** Negative**	0	0 ( 0.0%)	0 ( 0.0%)	8	6 (75.0%)	3 (37.5%)
** Fisher’s Exact Test** ***p*-value**		–-	–-		0.066	0.355
**Breast Cancer PR Status**						
** Positive**	0	0 (0.0%)	0 (0.0%)	52	20 (38.5%)	10 (19.2%)
** Negative**	0	0 (0.0%)	0 (0.0%)	18	10 (55.6%)	5 (27.8%)
** Fisher’s Exact Test** ***p*-value**		–-	–-		0.272	0.510
**Breast Cancer HER2-neu Status**						
** Positive**	0	0 (0.0%)	0 (0.0%)	16	10 (62.5%)	7 (43.8%)
** Equivocal**	0	0 (0.0%)	0 (0.0%)	6	1 (16.7%)	0 (0.0%)
** Negative**	0	0 (0.0%)	0 (0.0%)	41	15 (36.6%)	7 (17.1%)
** Unknown**	0	0 (0.0%)	0 (0.0%)	7	4 (57.1%)	1 (14.3%)
** Fisher’s Exact Test** ***p*-value**		–-	–-		0.158	0.092
**MBC Patient Disease Status**						
** Newly Diagnosed**	0	0 (0.0%)	0 (0.0%)	5	2 (40.0%)	0 (0.0%)
** Stable / Responding**	0	0 (0.0%)	0 (0.0%)	42	21 (50.0%)	12 (28.6%)
** Progressive / Recurrent**	0	0 (0.0%)	0 (0.0%)	23	7 (30.4%)	3 (13.0%)
** Fisher’s Exact Test** ***p*-value**		–-	–-		0.345	0.217

## Discussion

The ANG-008 study was designed to determine the proportion of HV subjects and MBC patients that have one or more CTCs harvested from a minimum of ≥ 5 mL of blood using the Parsortix^®^ PC1 System and identified using IF or cytology evaluations. Both downstream assays used in this study require deposition of the harvested cells onto a slide. As previously reported in the ANG-002 HOMING Clinical Study [27] and in Additional File 6, a substantial proportion of the cells harvested by the Parsortix^®^ PC1 System are not retained on the Cytospin™ slides when using cytocentrifugation. Unfortunately, this cell loss is an unavoidable limitation of any conventional centrifugation-based cytology slide preparation method, including the optimized method used in these studies. These observations must be kept in mind when evaluating the proportion of donors that have one or more observable tumor cells identified on their cytology slides. Given this large cell loss, it is possible that a larger proportion of MBC patients had CTCs present in their Parsortix^®^ PC1 System harvests, but these cells were simply not retained on the cytology slides. Other downstream analysis techniques (e.g. molecular evaluations) may be able to utilize cells captured by the Parsortix^®^ PC1 System that are harvested directly into a tube without subsequent manipulations of the harvested material that could potentially lead to cell losses.

Despite the significant cell losses caused by the cytocentrifugation method used for the preparation of the cytology slides, a significantly larger proportion of MBC patients had one or five or more cells identified as epithelial CTCs by IF (DAPI + , CD- and EpCAM + and/or CK + cells) compared to HV subjects. These results demonstrate that cells harvested by the Parsortix^®^ PC1 System can be evaluated using IF staining techniques and that only a very small proportion of the HV subjects had cells harvested by the Parsortix^®^ PC1 System that were identified as epithelial cells using IF evaluation. Interestingly, a high proportion of the CTCs identified did not express EpCAM, highlighting the limitations of using EpCAM-based approaches to capture CTCs. The significance of the circulating epithelial cells in the HV subjects is unknown, but other investigators using IF staining with similar targets have also shown a small proportion of subjects without disease having cells that appear to be of epithelial origin identified in their bloodstream [33]. It is interesting to note that the HV subject with 28 CTCs identified on their IF-stained cytology slide was pregnant at the time of their blood donation, with the intriguing possibility that the isolated CKs expressing cells could represent circulating fetal cells [34–36].

Epithelial CTC clusters were identified by IF in 56% of the MBC patients that had at least one cell classified as a CTC, indicating that the Parsortix^®^ PC1 System capturing process does not disrupt cells aggregates and biological adhesions, in contrast to other CTC detection apparatuses [32, 37–39]. As clusters of CTCs and clusters of CTCs with leukocytes are found to have differential biological features [32, 39], such as an enhanced survival and metastatic potential, the potential to harvest intact CTC clusters further expands the possible applications of the System for evaluation of prognosis, diagnosis and therapy of the metastatic cancer.

Additionally, by IF staining, it was also possible to further characterize the Parsortix^®^ PC1 Systems’ harvests. The filtration method used in the System was able to eliminate > 99.99% of the leukocytes present in the blood sample with a small number still retained. This is likely due to the varying sizes of leukocytes, with the larger and less compressible ones being captured in the critical gap of the separation cassette alongside CTCs, further highlighting the importance of using sensitive and specific downstream methods for discerning CTCs from leukocytes. Other non-typical circulating cells of interest were also identified. Based on their large size and a review of the literature [40–45], it is hypothesized that the smaller non-typical circulating cells are megakaryocytes that have released their platelets and, therefore, appeared naked/cytoplasm free while the larger cells are functioning megakaryocytes, which typically range up to 150 µm in diameter. A recently published non-interventional prospective study involving 59 patients with MBC showed that megakaryocytes (confirmed by immunocytochemistry staining with anti-CD61) were identified in Parsortix^®^ harvests of 52% of the MBC patients, corroborating the results presented in Additional File 4, showing that these cells are typically CD61 + , a marker (platelet glycoprotein IIIa) specific for the megakaryocytic lineage. Additionally, a similar weak positive correlation, like the one reported in our study, between the number of megakaryocytes and the number of CTCs (Pearson’s r = 0.416 (95% CI 0.179–0.608); p = 0.001) was reported in the literature [41]. Looking at correlations with demographic information, it was found that a statistically higher number of non-typical blood cells (when combining small and large) were present in donors receiving cytotoxic therapy. While it is not possible to accurately point out the cause of this increase, it was previously reported that cytotoxic drugs can alter thrombopoiesis within the bone marrow [46]. The clinical relevance of circulating megakaryocytes is still debated, with studies showing that high number of megakaryocytes correlated with poor survival in advanced prostate cancer [40]. This finding widens the scope of use of the Parsortix^®^ PC1 System, as the capture of megakaryocytes by the Parsortix^®^ PC1 System is justified by their large size, something not achievable using epitope-based CTC detection methods. Isolation of megakaryocytes by liquid biopsy may have a great impact on future research as more is learned about their role in cancer dissemination and correlation with CTCs, and clusters.

It was also possible for a pathologist to identify malignant cells (CTCs) by applying WG staining to the IF-stained slides, with a significantly larger proportion of MBC patients having one or five or more cells observed as being malignant on the WG-stained slides generated from their Parsortix^®^ PC1 System harvests compared to the HV subjects. These results demonstrate that cells harvested by the Parsortix^®^ PC1 System can be evaluated using another staining technique following IF staining; however, the discordance between the cells identified as epithelial CTCs by IF staining and the cells identified as being malignant on the same slides by WG staining should be noted. The cellular damage caused by the IF staining procedure is the most likely cause for the observed differences, but this needs to be investigated further. Alternatively, it is possible the discordance between the two methods is due to the lack of targeting of mesenchymal CTCs in the IF assay. These cells are not expected to express EpCAM and/or CK and would have been classified as DAPI + cells only, whereas the cytopathological review of the WG-stained cells would have allowed for their identification based on morphology.

Additional correlation analyses between CTCs and participants demographics showed that a significantly increased proportion of MBC patients had one or more cells classified as CTCs in port collected blood samples compared to the venous blood samples. This correlation was observed using both cytological evaluations and confirmed previously observed results obtained in the ANG-002 clinical study [27], where it was speculated that the increased CTC prevalence may be due to the fact that blood from a central port comes directly from the tumor without first filtering through additional capillary beds, while peripheral blood drawn from antecubital veins has likely circulated through both lung and peripheral capillaries after egressing from the tumor. Another possible hypothesis for this finding is that patients with a central port are usually receiving intravenous chemotherapy and, thus, may have a more aggressive disease compared to other MBC patients. The second correlation observed by IF was related to the use of pain medications. Due to the small sample size, nothing definitive can be concluded, however, it may be another association with more aggressive disease status. This correlation was not statistically significant when using a CTC threshold of ≥ 5 CTCs.

In addition to the previously detailed limitation about the use of cytocentrifugation, other limitations of this study were:

Set Volume of Blood Not Used: The ANG-008 clinical study specified that a minimum volume of blood needed to be available (≥ 5 mL) for the processing of each sample rather specifying that an exact volume of blood would be used for each sample. The primary reason for this was to reduce and minimize as much user intervention to the blood as possible (for example, decanting or pipetting an exact volume of blood into a separate vessel). Additionally, because the aim was not the enumeration of cells, but rather the capture and harvesting of cells for subsequent evaluation, it was felt that only a minimum volume of blood should be specified to demonstrate the feasibility of using different types of downstream analyses on the Parsortix^®^ System harvests. We recognize that the use of varying volumes of blood for each sample makes it more difficult to directly compare results between samples that used different volumes of blood. We also recognize that there is variability in the numbers of cells between different tubes (tube-to-tube variability).

Mesenchymal CTCs Not Assayed: The IF assay used in this study only included markers to target epithelial CTCs. However, it is known that tumor cells can undergo epithelial-to-mesenchymal transition (EMT) when entering the bloodstream to eventually establish distant metastases. Therefore, the inclusion of antibodies to target mesenchymal markers in the IF panel would maximize the information that can be obtained from each blood samples and potentially identify more clinically relevant CTCs for analysis. Nevertheless, while mesenchymal CTCs were not stained using the IF panel used in this study, it is possible to assume that EMT cells, with low/no expression of EpCAM and still retaining cytokeratins expression, were indeed detected. Additionally, as shown in Fig. 2, the percentage/number of nucleated unstained cells was comparable between HVs and MBC patients. While it is not possible to decipher the exact nature of these cells, the fact that comparable numbers are present in the healthy and patients’ cohorts indicates that the mesenchymal CTCs component was not very abundant in the patients included in this study, as compared to the already identified epithelial and EMT CTCs.

Clinical Utility Not Evaluated: The intention of the ANG-008 clinical study was to demonstrate that the Parsortix^®^ PC1 System could capture and harvest CTCs from the blood of metastatic breast cancer patients for subsequent analysis using IF and WG staining. The clinical utility of CTC enrichment in patients with MBC using the Parsortix^®^ PC1 System will need to be demonstrated in follow-up studies using validated downstream evaluation methods.

The IF staining process appeared to introduce a significant amount of cellular damage, making the cytopathological review of the WG-stained slides more difficult. Presumably this cellular damage was caused by the use of acetone as the cellular fixative as well as the IF staining process which required permeabilization of the cells due to the use of antibodies directed against intracellular targets. Fixation should be standardized to be able to successfully combine alternative staining methods on the same slide.

## Conclusion

This study demonstrated that the population of cells captured and harvested using the Parsortix^®^ PC1 System could be evaluated using IF and WG staining, and that a significantly larger proportion of MBC patients had one or five or more cells defined as being malignant compared to HV subjects. Interestingly, a majority of the cells classified as epithelial CTCs by IF did not express EpCAM, further highlighting limitations of using EpCAM-based approaches to capture CTCs. It was also possible to identify not only individual CTCs, but also clusters of CTCs and other non-typical circulating cells of interest, demonstrating that the Parsortix^®^ PC1 System could potentially be utilized to bridge the gap in CTC clusters analysis and further expand the understanding of metastasis dissemination that can be obtained from a liquid biopsy.

### Supplementary Information

Additional File 1. Mean analytical sensitivity and analytical specificity of the CK8/CK18/CK19-AF488 panel. (A) Dot plot shows mean ± SEM of the percentage analytical sensitivity of the CK panel in SKBR3 cells harvested from spiked blood of 20 healthy volunteers (N=20, mean=98%) separated through Parsortix^®^ instruments and percentage analytical specificity of the CK panel in Hs 578T cells harvested from spiked blood of 12 healthy donors (N=12, mean=100%) separated through Parsortix^®^ instruments. Only <1% (6/631) SKBR3 cells had a non-detectable CK signal indicating an overall analytical sensitivity of 99%. No Hs 578T cells had detectable CK signal, indicating an overall analytical specificity of 100%. (B) Representative image of a SKBR3 cell stained with the optimized panel. (C) Representative image of a Hs 578T cell stained with the optimized panel. Images were taken using a 10× objective on BioView Allegro Plus automated imaging system and are shown with 4× post imaging zoom. Merge colors: CD45/CD16/CD11b/CD61 (white), DAPI (blue), CK8/CK18/CK19 (green). Micron bar= 30 µm

Additional File 2. Analytical sensitivity and analytical specificity of EpCAM-AF555. (A) Dot plot shows mean ± SEM of EpCAM MFI (normalized for imaging exposure) in SKBR3, CaOV3, Hs 578T and WBCs. Line represents MFI value indicating detectable signal. Data were obtained from cancer cells harvested from spiked blood of healthy volunteers separated through Parsortix^®^ instruments. Analytical specificity was 100% as detected by the absence of detectable signal in Hs 578T cells (0/13) and in leukocytes (0/199). Analytical sensitivity was assessed in SKBR3 and CaOV3 cells with only 2/34 SKBR3 cells below the detectable threshold, indicating analytical sensitivity of 94%, and 0/21 CaOV3 cells below the detectable threshold, indicating analytical sensitivity of 100%.  (B) Representative images of EpCAM-AF555 staining in Hs 578T cells, CaOV3 cells, SKBR3 cells and WBCs. Images taken using a 10× objective on BioView Allegro Plus automated imaging system and are shown with 4× post imaging zoom. Merge colors: CD45/CD16/CD11b/CD61 (white), DAPI (blue), CK8/CK18/CK19 (green), EpCAM (red). Micron bar= 30 µm

Additional File 3. (A) Dot plot shows mean ± SEM of the percentage of harvested cells (excluding spiked cancer cells) stained by CD45 in 12 healthy volunteers’ harvests from Parsortix^®^ instruments. An average of 96% of the leukocytes found in healthy volunteers’ harvest samples expressed CD45. (B) Representative image of leukocytes stained by CD45 (APC, red) and DAPI (blue) and negative for CK8/CK18/CK19 (AF488, green) and EpCAM (AF555, orange). (C) Representative image of a SKBR3 cell stained by CK8/CK18/CK19 (AF488, green), EpCAM (AF555, orange) and DAPI (blue) and negative for CD45 (APC, red). Merge colors: CD45 (white), DAPI (blue), CK8/CK18/CK19 (green), EpCAM (red). Images taken with BioView Allegro Plus automated imaging system using a 10x objective lens and shown with 4× post imaging zoom. Micron bar= 30 µm

Additional File 4. (A) Dot plot shows mean ± SEM of the absolute number of CK+, EpCAM+/-, CDs- cells found when using CD45 alone, in combination with CD16/CD11b and in combination with CD16/CD11b/CD61, with and without user morphological evaluation on the identified cells in Parsortix^®^ harvests of metastatic breast cancer and healthy volunteer (HV) subjects. (B) Table shows percentage of samples with at least 1 CK+, EpCAM+/-, CD- cell. (C) Histogram shows mean ± SEM of the percentage of other non-typical circulating cells stained by CD61, CD16/CD11b or unstained.  Introduction of CD11b and CD16 into the CTCs exclusion panel reduced the level of unidentified cells of epithelial origin in HV samples from 70% to 50%, while no difference was observed in MBC samples. The use of CD61 further reduced the proportion of cells that were unidentified in HV subjects from 70% to 30%, while it did not affect positivity rate in MBC patients. CD61 was expressed by a large percentage of other non-typical blood cells, while no signal was observed in patients’ CTCs. Morphological evaluation further reduced unidentified epithelial events in healthy subjects to 15%. (D) Representative image of a CTC (top) and a non-typical circulating cell (bottom). Non-typical circulating cells have diameter of 20 – 80 µm and can be differentiated from CTCs based on CK signal distribution, cell size and nuclear/cytoplasmic ratio. Typically, in CTCs, the CK signal is localized in the cytoskeleton, in a ring-like pattern. CTCs are also characterized by misshapen nucleus, which appears brighter and more condensed compared to leukocytes’ nuclei. Non-typical blood cells have a large nucleus (>20 µm in diameter) with no/limited cytoplasm and present low CK expression localized as a diffused signal in the nuclear area with an overlap between DAPI and CK signals. Slides were imaged using a 10× objective on BioView Allegro Plus system and are shown with 4× post imaging zoom. Merge colors: CD61 (white), DAPI (blue), CK8/CK18/CK19 (green), EpCAM (red). Micron bar = 30 µm.

Additional File 5. Up to 15 contrived harvest sample slides containing WBCs and SKBR3 cells were stained with the final optimized panel. MFI (normalized for imaging exposure) of CK8/CK18/CK19 in AF488, EpCAM in AF555 and CD45/CD16/CD11b/CD61 in APC was assessed in positive and negative control cells to assess assay analytical sensitivity and specificity, respectively. (A) Histogram shows mean ± SEM of the MFI of each target in positive and negative cells. Green, orange and red lines show MFI values indicating detectable signal for CKs, EpCAM and CD45/CD16/CD11b/CD61, respectively. Mann-Whitney test was applied for significance between positive and negative cells, ****p≤0.0001. Analytical specificity and analytical sensitivity of CKs and CD markers was higher than 98%. EpCAM expression in SKBR3 and WBCs varied with a positivity rate of 80% and 6%, respectively. (B) Table shows number and percentage of SKBR3 cells (top row) and WBCs (bottom row) with detectable signal for CKs, EpCAM or CD markers.

Additional File 6. CellTracker^TM^ Orange prelabelled SKBR3 cells were spiked into K_2_EDTA tubes from 16 healthy volunteer subjects and separated through Parsortix^®^ instruments within 8 hours from draw. Samples were harvested into cytoslides and stained. Histogram shows mean ± SEM of the percentage of CellTracker™ Orange SKBR3 cells found in slide before and after staining compared to the number of cells captured in Parsortix^®^ separation cassette. Approximately 4% cell loss was observed following staining. Paired t-test applied; no statistically significant difference observed. The harvest processed combined with depositing cells onto cytoslides caused a mean cell loss of 62.3%.

## Data Availability

The datasets used and/or analysed during the current study are available from the corresponding author on reasonable request.

## Abbreviations

AF488: Alexa Fluor 488 dye
AF555: Alexa Fluor 555 dye
APC: Allophycocyanin
BMI: Body Mass Index
BSA: Body surface area
CBC: Complete Blood Count (CBC)
CFR: Code of Federal Regulations
CI: Confidence Interval
CTC: Circulating tumor cell
CK: Cytokeratin
DAPI: 4′,6-Diamidino-2-phenylindole
DNA: Deoxyribonucleic acid
EDTA: Ethylenediaminetetraacetic acid
EMT: Epithelial-Mesenchymal Transition
EpCAM: Epithelial Cell Adhesion Molecule
ER: Estrogen Receptor
FBS: Fetal bovine serum
FDA: Food and Drug Administration
GCP: Good Clinical Practice
HER2: Human epidermal growth factor receptor-2
HV: Healthy volunteer
ICH: International Conference on Harmonization
IF: Immunofluorescence
MBC: Metastatic breast cancer
mL: Milliliter
PBS: Phosphate Buffered Saline
PR: Progesterone Receptor
PC1: Parsortix^®^ PC1 instrument designation
RBC(s): Red Blood Cell(s)
RNA: Ribonucleic acid
SD: Standard Deviation
SEM: Standard Error of the Mean
SST: Serum-separating tubes
US: United States
WBC(s): White blood cell(s)
WG: Wright-Giemsa
µL: Microliter
µm: Micron or micrometer
