# Supplementary figures and images for: Identification of circulating tumor cells captured by the FDA-cleared Parsortix® PC1 system from the peripheral blood of metastatic breast cancer patients using immunofluorescence and cytopathological evaluations
Source: J Exp Clin Cancer Res. 2024 Aug 21;43:240. doi: 10.1186/s13046-024-03149-x (PMC11337573; doi:10.1186/s13046-024-03149-x)

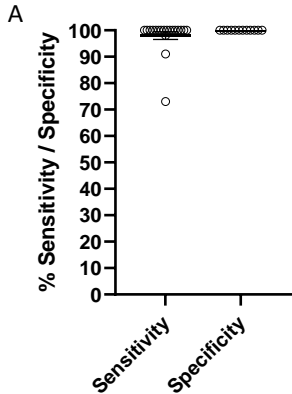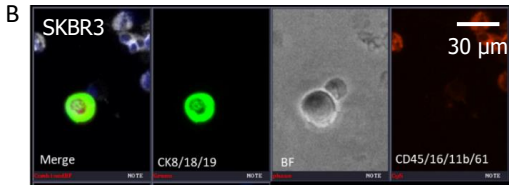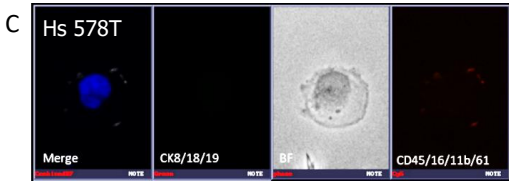

Supplement: Supplementary file 1 — Additional File 1. Mean analytical sensitivity and analytical specificity of the CK8/CK18/CK19-AF488 panel. (A) Dot plot shows mean ± SEM of the percentage analytical sensitivity of the CK panel in SKBR3 cells harvested from spiked blood of 20 healthy volunteers (N=20, mean=98%) separated through Parsortix® instruments and percentage analytical specificity of the CK panel in Hs 578T cells harvested from spiked blood of 12 healthy donors (N=12, mean=100%) separated through Parsortix® instruments. Only <1% (6/631) SKBR3 cells had a non-detectable CK signal indicating an overall analytical sensitivity of 99%. No Hs 578T cells had detectable CK signal, indicating an overall analytical specificity of 100%. (B) Representative image of a SKBR3 cell stained with the optimized panel. (C) Representative image of a Hs 578T cell stained with the optimized panel. Images were taken using a 10× objective on BioView Allegro Plus automated imaging system and are shown with 4× post imaging zoom. Merge colors: CD45/CD16/CD11b/CD61 (white), DAPI (blue), CK8/CK18/CK19 (green). Micron bar= 30 µm [file 13046_2024_3149_MOESM1_ESM.pdf]

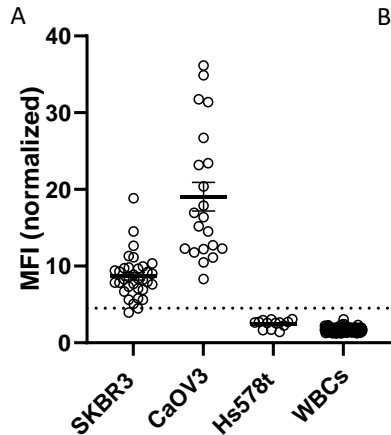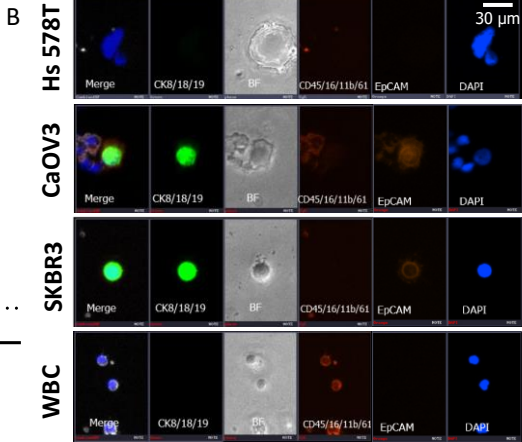

Supplement: Supplementary file 2 — Additional File 2. Analytical sensitivity and analytical specificity of EpCAM-AF555. (A) Dot plot shows mean ± SEM of EpCAM MFI (normalized for imaging exposure) in SKBR3, CaOV3, Hs 578T and WBCs. Line represents MFI value indicating detectable signal. Data were obtained from cancer cells harvested from spiked blood of healthy volunteers separated through Parsortix® instruments. Analytical specificity was 100% as detected by the absence of detectable signal in Hs 578T cells (0/13) and in leukocytes (0/199). Analytical sensitivity was assessed in SKBR3 and CaOV3 cells with only 2/34 SKBR3 cells below the detectable threshold, indicating analytical sensitivity of 94%, and 0/21 CaOV3 cells below the detectable threshold, indicating analytical sensitivity of 100%. (B) Representative images of EpCAM-AF555 staining in Hs 578T cells, CaOV3 cells, SKBR3 cells and WBCs. Images taken using a 10× objective on BioView Allegro Plus automated imaging system and are shown with 4× post imaging zoom. Merge colors: CD45/CD16/CD11b/CD61 (white), DAPI (blue), CK8/CK18/CK19 (green), EpCAM (red). Micron bar= 30 µm [file 13046_2024_3149_MOESM2_ESM.pdf]

A

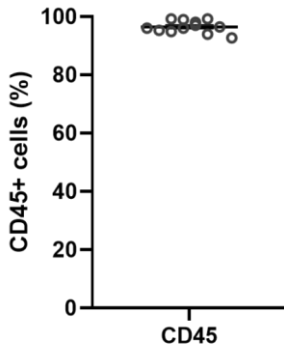

B

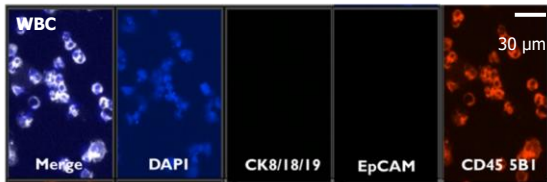

C

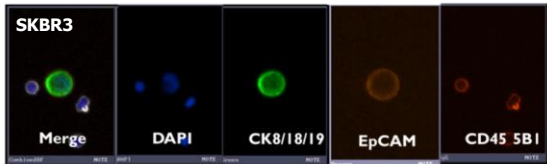

Supplement: Supplementary file 3 — Additional File 3. (A) Dot plot shows mean ± SEM of the percentage of harvested cells (excluding spiked cancer cells) stained by CD45 in 12 healthy volunteers’ harvests from Parsortix® instruments. An average of 96% of the leukocytes found in healthy volunteers’ harvest samples expressed CD45. (B) Representative image of leukocytes stained by CD45 (APC, red) and DAPI (blue) and negative for CK8/CK18/CK19 (AF488, green) and EpCAM (AF555, orange). (C) Representative image of a SKBR3 cell stained by CK8/CK18/CK19 (AF488, green), EpCAM (AF555, orange) and DAPI (blue) and negative for CD45 (APC, red). Merge colors: CD45 (white), DAPI (blue), CK8/CK18/CK19 (green), EpCAM (red). Images taken with BioView Allegro Plus automated imaging system using a 10x objective lens and shown with 4× post imaging zoom. Micron bar= 30 µm [file 13046_2024_3149_MOESM3_ESM.pdf]

**A**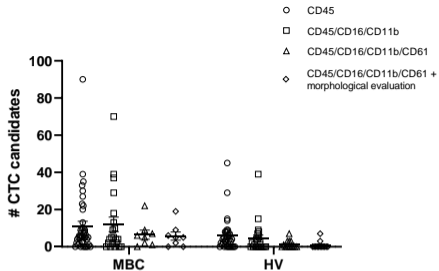**B**

|              | CD Markers used: |                 |                      |                                   |
|--------------|------------------|-----------------|----------------------|-----------------------------------|
| % Positivity | CD45 only        | CD45/CD16/CD11b | CD45/CD16/CD11b/CD61 | CD45/CD16/CD11b/CD61 + morphology |
| MBC          | 83%              | 76%             | 87%                  | 75%                               |
| HV           | 70%              | 50%             | 38%                  | 15%                               |

**C**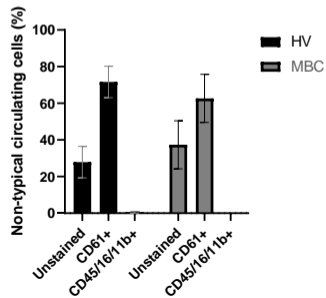**D**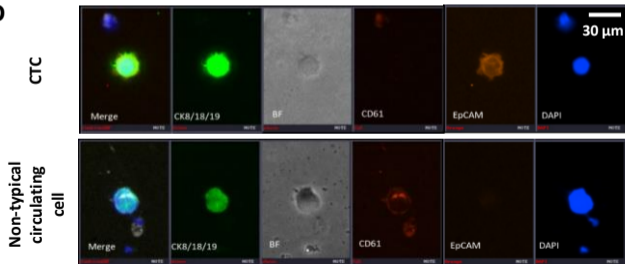

Supplement: Supplementary file 4 — Additional File 4. (A) Dot plot shows mean ± SEM of the absolute number of CK+, EpCAM+/-, CDs- cells found when using CD45 alone, in combination with CD16/CD11b and in combination with CD16/CD11b/CD61, with and without user morphological evaluation on the identified cells in Parsortix® harvests of metastatic breast cancer and healthy volunteer (HV) subjects. (B) Table shows percentage of samples with at least 1 CK+, EpCAM+/-, CD- cell. (C) Histogram shows mean ± SEM of the percentage of other non-typical circulating cells stained by CD61, CD16/CD11b or unstained. Introduction of CD11b and CD16 into the CTCs exclusion panel reduced the level of unidentified cells of epithelial origin in HV samples from 70% to 50%, while no difference was observed in MBC samples. The use of CD61 further reduced the proportion of cells that were unidentified in HV subjects from 70% to 30%, while it did not affect positivity rate in MBC patients. CD61 was expressed by a large percentage of other non-typical blood cells, while no signal was observed in patients’ CTCs. Morphological evaluation further reduced unidentified epithelial events in healthy subjects to 15%. (D) Representative image of a CTC (top) and a non-typical circulating cell (bottom). Non-typical circulating cells have diameter of 20 – 80 µm and can be differentiated from CTCs based on CK signal distribution, cell size and nuclear/cytoplasmic ratio. Typically, in CTCs, the CK signal is localized in the cytoskeleton, in a ring-like pattern. CTCs are also characterized by misshapen nucleus, which appears brighter and more condensed compared to leukocytes’ nuclei. Non-typical blood cells have a large nucleus (>20 µm in diameter) with no/limited cytoplasm and present low CK expression localized as a diffused signal in the nuclear area with an overlap between DAPI and CK signals. Slides were imaged using a 10× objective on BioView Allegro Plus system and are shown with 4× post imaging zoom. Merge colors: CD61 (white), DAPI (blu [file 13046_2024_3149_MOESM4_ESM.pdf]

A

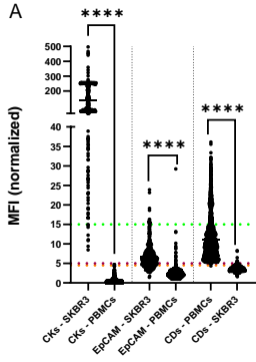

B

|       | CKs+            | EpCAM+          | CDs+               |
|-------|-----------------|-----------------|--------------------|
| SKBR3 | 407/413:<br>99% | 336/413:<br>81% | 2580/2580:<br>100% |
| WBCs  | 0/2580:<br>0%   | 15/2580:<br>6%  | 10/630:<br>2%      |

Supplement: Supplementary file 5 — Additional File 5. Up to 15 contrived harvest sample slides containing WBCs and SKBR3 cells were stained with the final optimized panel. MFI (normalized for imaging exposure) of CK8/CK18/CK19 in AF488, EpCAM in AF555 and CD45/CD16/CD11b/CD61 in APC was assessed in positive and negative control cells to assess assay analytical sensitivity and specificity, respectively. (A) Histogram shows mean ± SEM of the MFI of each target in positive and negative cells. Green, orange and red lines show MFI values indicating detectable signal for CKs, EpCAM and CD45/CD16/CD11b/CD61, respectively. Mann-Whitney test was applied for significance between positive and negative cells, ****p≤0.0001. Analytical specificity and analytical sensitivity of CKs and CD markers was higher than 98%. EpCAM expression in SKBR3 and WBCs varied with a positivity rate of 80% and 6%, respectively. (B) Table shows number and percentage of SKBR3 cells (top row) and WBCs (bottom row) with detectable signal for CKs, EpCAM or CD markers. [file 13046_2024_3149_MOESM5_ESM.pdf]

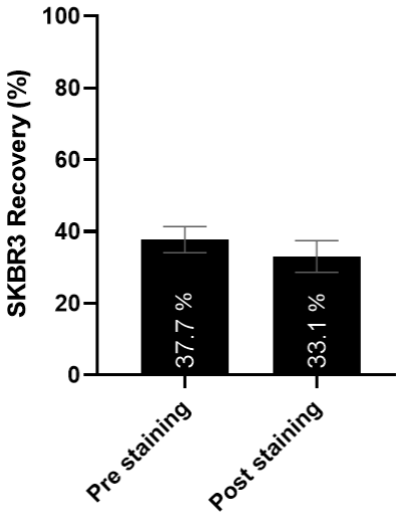

Supplement: Supplementary file 6 — Additional File 6. CellTrackerTM Orange prelabelled SKBR3 cells were spiked into K2EDTA tubes from 16 healthy volunteer subjects and separated through Parsortix® instruments within 8 hours from draw. Samples were harvested into cytoslides and stained. Histogram shows mean ± SEM of the percentage of CellTracker™ Orange SKBR3 cells found in slide before and after staining compared to the number of cells captured in Parsortix® separation cassette. Approximately 4% cell loss was observed following staining. Paired t-test applied; no statistically significant difference observed. The harvest processed combined with depositing cells onto cytoslides caused a mean cell loss of 62.3%. [file 13046_2024_3149_MOESM6_ESM.pdf]
